# Supplementary material for: A workflow for sizing oligomeric biomolecules based on cryo single molecule localization microscopy
Source: PLoS One. 2021 Jan 20;16(1):e0245693. doi: 10.1371/journal.pone.0245693 (PMC7817001; doi:10.1371/journal.pone.0245693)
Supplement: S1 File — (PDF) [file pone.0245693.s001.pdf]

S1 File

A workflow for sizing oligomeric biomolecules based on cryo single molecule localization microscopy

Magdalena C. Schneider[1+], Roger Telschow[2+], Gwenael Mercier[2], Montserrat López-Martinez[1], Otmar Scherzer[2*], and Gerhard J. Schütz[1*]

[1]Institute of Applied Physics, TU Wien, Vienna, Austria
[2]Faculty of Mathematics, University of Vienna, Vienna, Austria
[*]otmar.scherzer@univie.ac.at (OS), schuetz@iap.tuwien.ac.at (GJS)
[+]these authors contributed equally to this work

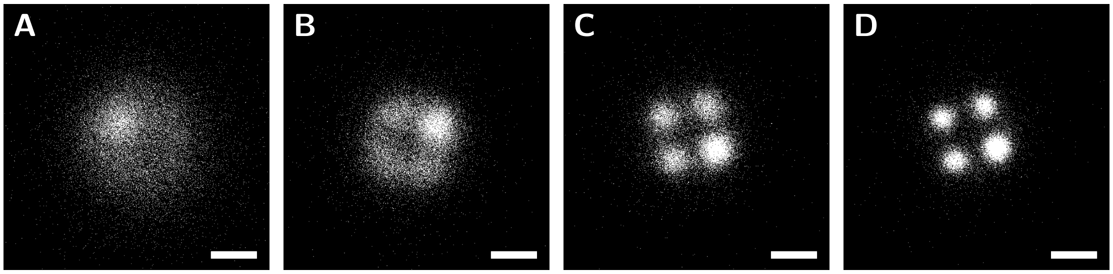

Figure S1

Reconstruction with template-free method. Data analyzed with the template-free method published by Heydarian et al.[1] For the analysis we simulated 1000 tetramers, with a side length of 5 nm, assuming $N_{\max} = 10^4$ photons **(a)**, $N_{\max} = 3 \cdot 10^4$ photons **(b)**, $N_{\max} = 5 \cdot 10^4$ photons **(c)** and $N_{\max} = 10^5$ photons **(d)**. Scale bars: 5 nm.

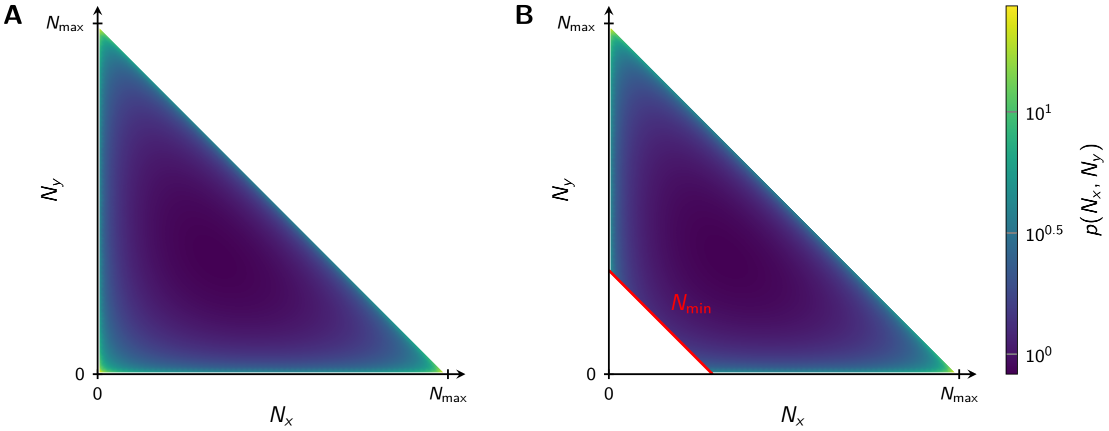

Figure S2

Probability density for $(\mathbf{N_x}, \mathbf{N_y})$. Dipole orientations are assumed to be homogeneously distributed. Naturally, $N_x + N_y > N_{\max}$ cannot occur. Panel **(a)** shows the full probability distribution according to Eq. (4) from the *Methods*, panel **(b)** a truncated distribution, which accounts for the user-defined detection threshold $N_{\min}$. For the calculation of panel (b) we assumed $N_{\max} = 10^4$ photons and background noise $b = 300$, yielding $N_{\min} = 2960$.

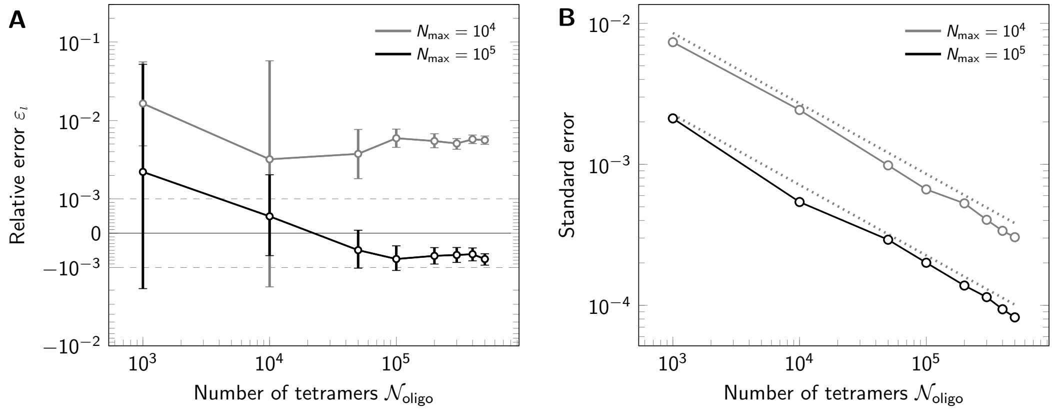

Figure S3

Influence of number of available oligomers. Effect of varying numbers of simulated tetramers $\mathcal{N}_{\text{oligo}}$ on the relative error ε_l **(a)** and its standard error **(b)**. Results are shown both for a maximum photon number $N_{\text{max}} = 10^4$ (gray) and $N_{\text{max}} = 10^5$ (black). Positive and negative relative errors represent overestimation and underestimation, respectively. The dashed line in panel (b) indicates $\mathcal{N}_{\text{oligo}}^{-1/2}$-dependence. Error bars indicate the 95% confidence intervals.

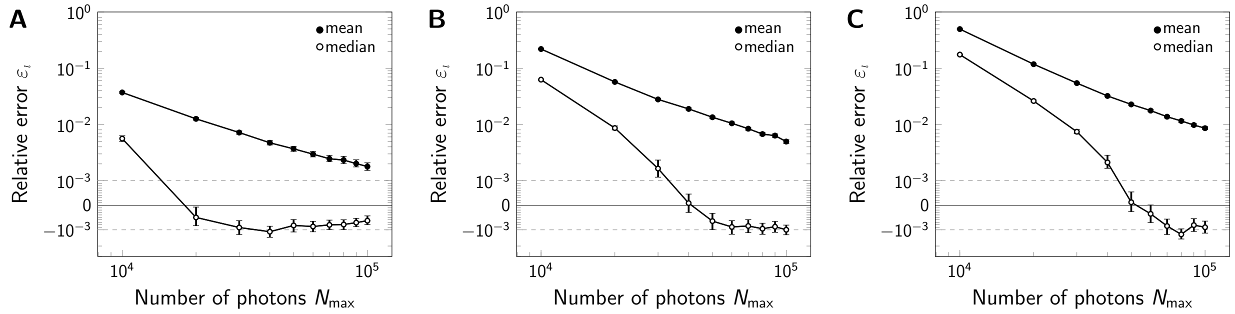

Figure S4

Influence of signal brightness for different levels of background noise. Relative error ε_l for varying maximum photon number N_{max}, including background noise of $b = 0$ **(a)**, $b = 100$ **(b)** and $b = 300$ **(c)**. For each data point 500 000 tetramers (side length 5 nm) were simulated. In both panels we compared the analysis via the mean (full symbols) and median (open symbols). Positive and negative relative errors represent overestimation and underestimation, respectively. Error bars indicate the 95% confidence intervals.

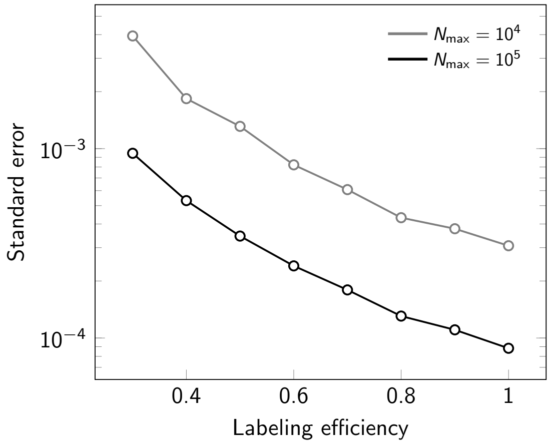

Figure S5

Standard error of the median for varying labeling efficiency. Assumed photon numbers were $N_{max} = 10^4$ (light line) and $N_{max} = 10^5$ (dark line). For each data point 500 000 tetramers were simulated.

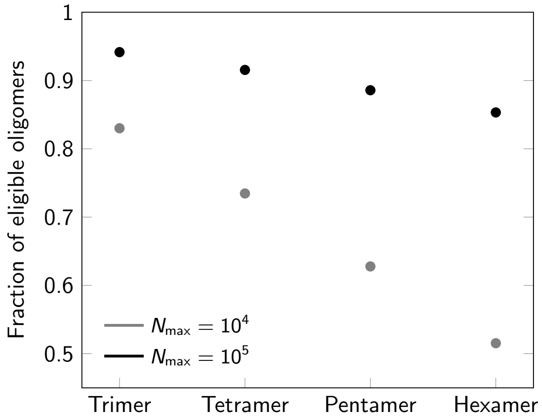

Figure S6

Percentage of eligible oligomers for varying degree of oligomerization. Assumed photon numbers were $N_{max} = 10^4$ (gray) and $N_{max} = 10^5$ (black).

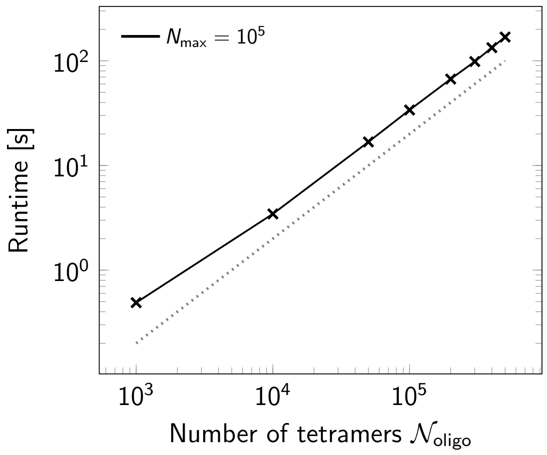

Figure S7
Runtime scaling. Analysis of runtime for varying number $\mathcal{N}_{\text{oligo}}$ of simulated tetramers, assuming $N_{\text{max}} = 10^5$ photons. The dashed line indicates linear complexity.

Supplementary Note 1 — Probability density of $(N_{\mathbf{x}}, N_{\mathbf{y}})$

In the following we calculate the probability density for detecting (N_x, N_y) photons for dipole moments distributed randomly on a sphere. To ensure a bijective functional relationship between (N_x, N_y) and (θ, ϕ) as specified by Eq. (2) and (3) in the subsection *Simulations* in the *Methods* of the manuscript, we confine the analysis to the octant defined by (θ, ϕ) in the intervals $[0, \frac{\pi}{2}] \times [0, \frac{\pi}{2}]$. The probability densities for θ and ϕ are given by

$$\rho_{\text{az.}}(\theta) = \begin{cases} \cos(\theta) & \text{for } \theta \in [0, \frac{\pi}{2}], \\ 0 & \text{otherwise,} \end{cases}$$

$$\rho_{\text{el.}}(\phi) = \begin{cases} \frac{2}{\pi} & \text{for } \phi \in [0, \frac{\pi}{2}], \\ 0 & \text{otherwise,} \end{cases}$$

the joint probability density for (θ, ϕ) by

$$\rho_{\text{dipole}}(\theta, \phi) = \begin{cases} \frac{2}{\pi}\cos(\theta) & \text{for } \theta \in [0, \frac{\pi}{2}], \phi \in [0, \frac{\pi}{2}], \\ 0 & \text{otherwise.} \end{cases}$$

Inverting equations (2) and (3) from the *Methods* yields

$$\theta = \arccos\left(\sqrt{\frac{N_\text{x} + N_\text{y}}{N_\text{max}}}\right), \qquad \phi = \arccos\left(\sqrt{\frac{N_\text{x}}{N_\text{x} + N_\text{y}}}\right)$$

and for the joint probability density of the detected number of photons (N_x, N_y)

$$\begin{aligned} \rho_{\text{phot.}}(N_x, N_y) &= \rho_{\text{dipole}}\left(\arccos\left(\sqrt{\frac{N_\text{x} + N_\text{y}}{N_\text{max}}}\right), \arccos\left(\sqrt{\frac{N_\text{x}}{N_\text{x} + N_\text{y}}}\right)\right) \cdot \left|\frac{\partial(\theta, \phi)}{\partial(N_x, N_y)}\right| \\ &= \frac{2}{\pi}\sqrt{\frac{N_\text{x} + N_\text{y}}{N_\text{max}}} \cdot \left|\frac{\partial(\theta, \phi)}{\partial(N_x, N_y)}\right|. \end{aligned} \tag{1}$$

The Jacobian yields

$$\begin{aligned} \left|\frac{\partial(\theta, \phi)}{\partial(N_x, N_y)}\right| &= \left|\begin{bmatrix} -\frac{1}{2}\Big((N_\text{x} + N_\text{y})(N_\text{max} - N_\text{x} - N_\text{y})\Big)^{-\frac{1}{2}} & -\frac{1}{2}\Big((N_\text{x} + N_\text{y})(N_\text{max} - N_\text{x} - N_\text{y})\Big)^{-\frac{1}{2}} \\ -\frac{1}{2}\frac{1}{N_\text{x}+N_\text{y}}\sqrt{\frac{N_\text{y}}{N_\text{x}}} & \frac{1}{2}\frac{1}{N_\text{x}+N_\text{y}}\sqrt{\frac{N_\text{x}}{N_\text{y}}} \end{bmatrix}\right| \\ &= \frac{1}{4}\Big(N_\text{x}N_\text{y}(N_\text{x} + N_\text{y})(N_\text{max} - N_\text{x} - N_\text{y})\Big)^{-\frac{1}{2}}. \end{aligned}$$

Inserting into Equation (**??**) yields the joint probability density of (N_x, N_y)

$$\rho_{\text{phot.}}(N_x, N_y) = \begin{cases} \frac{1}{2\pi}\Big(N_\text{max}N_\text{x}N_\text{y}(N_\text{max} - N_\text{x} - N_\text{y})\Big)^{-\frac{1}{2}} & \text{for } N_x, N_y \geq 0,\ N_x + N_y \leq N_\text{max}, \\ 0 & \text{otherwise.} \end{cases}$$

References

1. Heydarian, H. *et al.* Template-free 2D particle fusion in localization microscopy. *Nat. Meth.* **15**, 781–784, DOI: 10.1038/s41592-018-0136-6 (2018).
