# Supplementary material for: A workflow for sizing oligomeric biomolecules based on cryo single molecule localization microscopy
Source: PLoS One. 2021 Jan 20;16(1):e0245693. doi: 10.1371/journal.pone.0245693 (PMC7817001; doi:10.1371/journal.pone.0245693)
Supplement: Attachment — Submitted filename: Reviewcomments.pdf [file pone.0245693.s003.pdf]

Schneider et al have discusses a data analysis strategy for cryo-SMLM experiments. In this work they make use of polarization of the fluorophore to localize single dye molecule.

Major concerns

- The following points need clarification:
  - **Characteristics of dipole at low temperatures:** Considering the importance of the dipole being fixed at cryogenic temperatures, insufficient information has been provided on the stability of the dipole orientation at cryogenic temperatures. Authors should provide references.
  - **External factors that could affect signal:** Effects of variations in the orientation of the oligomer, photo bleaching, and the local environment (solvent) on the intensity of the dipole signal haven't been factored into the model. These points should be discussed.
- Line 278 onwards, authors point out the requirements that need to be fulfilled for their technique to be adopted. Can they quantify these requirements?
  - Point 5 "The mutual distance between different oligomers should be large" – what does large mean here?
  - Point 6 "Oligomerization should occur only in a plane perpendicular to the optical axis." How flexible is their method is if the oligomerization plane is tilted? A quantification on "tilt vs error" measurements/ simulation hasn't been shown.
  - This also raises another concern: how much error does sample preparation cause, for example by pushing fluorophores out of the optical plane?
  - Authors provide a generic solution to reject sample that do not fit author's criterion. My primary concern is that this would lead to rejection of valuable datasets and bias the analysis. Perhaps they could provide some discussion on this issue.

Minor concerns

- In Fig3 - why is there a significant difference between the mean and median. Explain how underestimation and overestimation of size depend on photon count? How are tetramer numbers expected to affect the simulations?
- It seems impractical to image 500,000 tetramers in laboratory settings. This is a concern as the standard error cannot be reduced without a large sampling, which limits the applicability of this method.
- In Fig 3A, is the error bar too small to visualize in some cases? Are these errors or standard deviations? Please comment on the significance here.
- Line 64 - Authors mention that "none of these methods attempt to assign localizations to specific dye molecules". However, in reference [8] the authors say "…our measurement resolves the two labels within a single protein". Can the authors clarify how the localization of specific dye molecules improves on that work?
