# Supplementary material for: A workflow for sizing oligomeric biomolecules based on cryo single molecule localization microscopy
Source: PLoS One. 2021 Jan 20;16(1):e0245693. doi: 10.1371/journal.pone.0245693 (PMC7817001; doi:10.1371/journal.pone.0245693)
Supplement: Attachment — Submitted filename: Response to Reviewers.pdf [file pone.0245693.s004.pdf]

We thank both reviewers for their thoughtful inputs to our manuscript. In the revised version we addressed all issues raised. Below, you find a point-by-point reply, including references to the changes in the manuscript. Note that line numbers refer to the manuscript with tracked changes.

In addition, we spotted a mistake in our previous submission, which we corrected in the revised manuscript. In the original text, we mentioned two different methods for the correction of the localization bias, when performing circular fits, which we termed Method 1 and Method 2. Method 1 appeared superior for large oligomers, Method 2 for small oligomers. We found out, however, that there was an error in the code for calculating the results via Method 2; in the correct version, it yields virtually identical results as Method 1. We hence decided to remove the description of Method 2 from the text, and we removed the according dashed lines from Fig. 5. Also Supplementary Fig. S6, which was only needed to substantiate the discrimination between the two Methods, was removed. Importantly, no conclusion of the paper is effected by this mistake.

Referee 1

<u>Major concerns</u>

The following points need clarification:

<u>Question:</u> Characteristics of dipole at low temperatures: Considering the importance of the dipole being fixed at cryogenic temperatures, insufficient information has been provided on the stability of the dipole orientation at cryogenic temperatures. Authors should provide references.

Answer:
In the revised introduction section (line 70), we added a reference to the paper by Weisenburger et al. (Nature Methods 14: 141-144), who did not observe any detectable alterations of the single molecule brightness levels within 100 minutes, when experiments were performed at cryogenic temperatures. In that paper, samples were illuminated by circularly polarized light. Hence, residual rotational motion of the fluorophores would have resulted in alterations of the inclination angle of the dipole, thereby changing the probability of photon absorption.

Given the fact that for cryo-EM, samples are routinely stored over months at liquid nitrogen temperatures without detectable alterations of protein structures, much longer stability of the dyes' orientation can be expected. Since we did not find any thorough investigation of this structural preservation, however, we reverted from discussing this aspect.

<u>Question:</u> External factors that could affect signal: Effects of variations in the orientation of the oligomer, photo bleaching, and the local environment (solvent) on the intensity of the dipole signal haven't been factored into the model. These points should be discussed.

Answer:
The reviewer mentions two important aspects: First, the oligomeric structure may be not coplanar with the plane of focus. We addressed this aspect below when we discuss the reviewer's question about tilt angles.

Second, the local environment may well affect the single molecule brightness. This influence, however, would not deteriorate the performance of our method. On the contrary, it would provide an additional means for discriminating blinks from different dye molecules, based on additional differences in the recorded single molecule brightness. We added a statement in the discussion section (lines 291-294).

Photobleaching limits the number of counts per dye molecule. In the extreme case, a particular protomer may be bleached before being detected. This scenario, however, is unproblematic for the size estimation, since only oligomers which yield n distinct groups of localizations are taken for further analysis (eligibility criterion, line 165). For our simulations, we used log-normally distributed blinking characteristics, which match experimental data at room temperature. At low temperatures, however, one may expect reduced photobleaching kinetics, thereby further increasing the number of localizations per dye molecule. In consequence, size estimates can be expected to become even more precise. We added a statement in lines 295-298 of the discussion section.

Line 278 onwards, authors point out the requirements that need to be fulfilled for their technique to be adopted. Can they quantify these requirements?

<u>Question:</u> Point 5 "The mutual distance between different oligomers should be large" – what does large mean here?

Answer: We specified the distance on line 337. It reads now $d \gg R + \Delta x$.

<u>Question:</u> Point 6 "Oligomerization should occur only in a plane perpendicular to the optical axis." How flexible is their method is if the oligomerization plane is tilted? A quantification on "tilt vs error" measurements/ simulation hasn't been shown.

Answer: We thank the reviewer for this valid point. In the revised version of our manuscript we included the analysis of tetramers located on a surface, which is tilted by an angle between 0 and 40 degrees. Expectedly, the circular fit underestimates the size of the oligomers. In the new Fig. 7, we show that up to 10 degrees tilt the relative errors stay below 1%. Surprisingly, even massive tilts of 40 degrees only lead to a 10% underestimation of the obtained tetramer size. The data are described in lines 264-269. In addition, we modified the discussion section (lines 343-346) and described the tilting procedure in the methods section (lines 412-415).

<u>Question:</u> **This also raises another concern: how much error does sample preparation cause, for example by pushing fluorophores out of the optical plane?**

Answer: Indeed, all fluorophores should be located in the focal plane. Due to the fixed orientation of the dipoles, the corresponding PSF will generally be tilted against the optical axis. Even slight defocusing may hence substantially displace the obtained localizations from the true fluorophore position. Recently, two methods were proposed to solve this problem: Azimuthal filtering, introduced by the Moerner lab (Backlund et al., 2016), or polarization-resolved imaging, introduced by the group of Jörg Enderlein (Nevskyi et al., 2020). We discussed this issue in the revised manuscript in lines 301-306.

<u>Question:</u> Authors provide a generic solution to reject sample that do not fit author's criterion. My primary concern is that this would lead to rejection of valuable datasets and bias the analysis. Perhaps they could provide some discussion on this issue.

Answer: We partially agree with the reviewer. While it is unlikely that the rejection of incompletely labelled oligomers generates a bias to the size estimates, it may well be that they contain information which would help to improve the precision. Essentially, the number of analyzable oligomers would increase in Suppl. Fig. 3B, leading to a decrease in the standard error of size estimation.

The criterion, however, is extremely helpful to reject scenarios, where two or more groups of localizations overlap and hence would be interpreted as one spurious position at the weighted average

of the detected localizations. This has not been mentioned in the original manuscript, and therefore was added in lines 166-168.

Minor concerns

Question: ***In Fig3 - why is there a significant difference between the mean and median.***

Answer: Naturally, the distribution of estimated side lengths is limited to positive values and therefore, is slightly positively skewed (see line 184). In such cases, there is a difference between mean and median.

Question: ***Explain how underestimation and overestimation of size depend on photon count?***

At this stage we do not know the answer to this question. According to Suppl. Fig. 4, increasing background noise alters the transition from overestimation to underestimation as a function of photon count. Likely, this has to do with taking the median value as the estimator of the oligomer size. We would like to emphasize, however, that the deviations from the true oligomer size are below 0.1%, which we consider to be absolutely acceptable for most practical applications. For example, for a 5nm tetramer the results would be off by 0.005nm.

Question: ***How are tetramer numbers expected to affect the simulations?***

Answer: This information has already been provided in Suppl. Fig. 3 and mentioned in lines 193-197.

Question: ***It seems impractical to image 500,000 tetramers in laboratory settings. This is a concern as the standard error cannot be reduced without a large sampling, which limits the applicability of this method.***

Answer: For our manuscript, we performed simulations on a large number of oligomers, in order to depict the trends and dependencies adequately. In a practical setting, however, it suffices to record substantially fewer structures as shown also in Suppl. Fig. 3. For example, assuming $N_{max}=10^5$ photons (which is absolutely realistic under cryogenic conditions), it is sufficient to record 1000 tetramers for a standard error of 0.02.

Question: ***In Fig 3A, is the error bar too small to visualize in some cases? Are these errors or standard deviations? Please comment on the significance here.***

Answer: The assumption is correct, the error bars were too small to see in some cases. This question of the reviewer prompted us to display the figures in a different way. Instead of plotting the standard error of the mean (or median), we show now the 95% confidence intervals, which allow for a direct interpretation in terms of statistical significance: If the 95% confidence intervals do not overlap, there is a significant difference at a significance level of $\alpha=0.05$. We adapted the section describing the error bars in the methods section (lines 629-630).

In addition, we found a better way of displaying over- and underestimation of results in the logarithmic plots, using a symmetric logarithmic display, which shows positive and negative relative errors on the positive and negative y-axis, respectively. Relative errors $|\varepsilon| < 10^{-3}$ are shown on a linear scale (lines 203-206).

<u>Question:</u> Line 64 - Authors mention that "none of these methods attempt to assign localizations to specific dye molecules". However, in reference [8] the authors say "…our measurement resolves the two labels within a single protein". Can the authors clarify how the localization of specific dye molecules improves on that work?

Answer: Indeed, Weisenburger et al. utilize characteristic brightness levels of single molecule signals to assign localizations. As noted by the authors, however, this approach becomes instable when four or more dye molecules are present on the very same complex. Essentially, our approach of using polarization information adds a second dimension to the clustering problem of brightness levels (see our Fig. 1B). For example, when projected on the x-axis, the blue and the green cloud of localizations would be impossible to discriminate, whereas in a two-dimensional representation they are clearly separated. We rephrased our statement in the introduction section (line 64) accordingly.

Referee 2

<u>Question:</u> Taking together, the manuscript describes an interesting approach and useful theoretical study. I have, however, one concern, which the authors should address before publication. I am wondering how energy transfer pathways (energy hopping, single-singlet annihilation, etc.) between nanometer separated dipoles influences or in the worst case scenario prevents the localization of all emitters attached to the regular oligomeric structure. While I believe these processes are of minor importance for distances larger than 5 nm they certainly complicate data analysis for shorter distances.

Answer: We thank the reviewer for bringing up this point. In theory, inter-fluorophore energy transfer could indeed spread the excitation, thereby generating an image consisting of an average of multiple point-spread functions, representing the individual emitters. In practice, however, we do not consider this to be too problematic. In our concept, we consider fluorophores to show long-lived transitions to dark (likely triplet) states at cryogenic temperatures. At least for some organic dyes such as Atto647N (Weisenburger et al., 2017) or JF525 (Hoffman et al., 2020) this assumption is justified. As long as only one dye molecule per oligomer gets cycled between S_0 and S_1, the resulting point spread function corresponds to the active dye. If the excitation of the active dye stimulated the reverse intersystem-crossing of a dark dye (Tinnefeld et al., 2003), however, one would observe the abrupt appearance of a second signal overlapping with the first signal, corrupting the analysis. Such events can be filtered out, as they show an abrupt change in the polarization during the time sequence. Of note, such a mechanism was not observed in the pioneering paper by Weisenburger et al., in which inter-dye distances of appr. 1 nm could be determined (Weisenburger et al., 2017). We added a bullet point to the discussion section (lines 319-326).

References

Backlund, M.P., A. Arbabi, P.N. Petrov, E. Arbabi, S. Saurabh, A. Faraon, and W.E. Moerner. 2016. Removing orientation-induced localization biases in single-molecule microscopy using a broadband metasurface mask. *Nature Photonics*. 10:459-462.

Hoffman, D.P., G. Shtengel, C.S. Xu, K.R. Campbell, M. Freeman, L. Wang, D.E. Milkie, H.A. Pasolli, N. Iyer, J.A. Bogovic, D.R. Stabley, A. Shirinifard, S. Pang, D. Peale, K. Schaefer, W. Pomp, C.-L. Chang, J. Lippincott-Schwartz, T. Kirchhausen, D.J. Solecki, E. Betzig, and H.F. Hess. 2020. Correlative three-dimensional super-resolution and block-face electron microscopy of whole vitreously frozen cells. *Science*. 367:eaaz5357.

Nevskyi, O., R. Tsukanov, I. Gregor, N. Karedla, and J. Enderlein. 2020. Fluorescence polarization filtering for accurate single molecule localization. *APL Photonics*. 5:061302.

Tinnefeld, P., V. Buschmann, K.D. Weston, and M. Sauer. 2003. Direct Observation of Collective Blinking and Energy Transfer in a Bichromophoric System. *The Journal of Physical Chemistry A*. 107:323-327.

Weisenburger, S., D. Boening, B. Schomburg, K. Giller, S. Becker, C. Griesinger, and V. Sandoghdar. 2017. Cryogenic optical localization provides 3D protein structure data with Angstrom resolution. *Nat Meth*. 14:141-144.
